# “When She Says Daddy”: Black Fathers’ Recidivism following Reentry from Jail

**DOI:** 10.3390/ijerph19063518

**Published:** 2022-03-16

**Authors:** Alvin Thomas, Jennifer Clare Wirth, Julie Poehlmann-Tynan, David J. Pate

**Affiliations:** 1 Human Development and Family Studies Department, School of Human Ecology, University of Wisconsin-Madison, Madison, WI 53706, USA; jcwirth@wisc.edu (J.C.W.); julie.poehlmanntynan@wisc.edu (J.P.-T.); 2 Helen Bader School of Social Welfare, University of Wisconsin-Milwaukee, Milwaukee, WI 53201, USA

**Keywords:** children, family, fathers, jail, recidivism

## Abstract

We report on the findings of a mixed methods longitudinal study of 84 African American fathers of young children who were enrolled into the study during the father’s jail stay. Participants were assessed using interviews, self-report measures, and administrative records on frequency of father–child contact, father–caregiver relationship quality, family support, paternal pre-incarceration employment, fathers’ plans to live with the child upon reentry, history of substance abuse, and new convictions one year following release from jail. Qualitative analysis revealed three primary identities of fathers during incarceration: father as nurturer, father as protector, and father as provider. Qualitative analysis of interview data detailed the ways in which the context of incarceration and the presence of the criminal justice system interacts with these identities to impact family structure, parent–child visits, plans for release, and motivation for desistance. Quantitative analysis indicated heterogeneity among fathers, with links between parent–child contact and desistance conditional on fathers’ plans for coresidence with children as well as family support and relationship quality. Taken together, the findings highlight the strengths of African American fathers and their families despite the risks associated with incarceration, including the importance of family support and children as motivation for desistance. The results have implications for how the justice system weighs the bidirectional influences of fathers and families.

## 1. Introduction

The United States has been in a period of mass incarceration for several decades, with nearly 2.1 million individuals locked in prisons or jails at year end 2019 [1] and more than 10 million admissions to jails each year [2]. Incarceration is unequally distributed in the U.S., with Black men, especially those who are poor and with low education, disproportionately affected [3,4]. For example, at year-end 2017, the imprisonment rate for sentenced Black men, 2336 per 100,000 Black male U.S. residents, was almost six times that of sentenced White men, 397 per 100,000 White male U.S. residents [5]. Almost half of incarcerated men are fathers of minor children, with higher percentages among Black and Latino incarcerated men than White incarcerated men [6]. For fathers involved in the criminal justice system, recidivism remains a significant challenge as they seek to reintegrate into their families, places of employment, and communities following incarceration. In the present mixed methods longitudinal convergent study, we examined 1-year recidivism in Black fathers of young children in relation to their contact with a focal child, relationships with children’s caregivers, and family support, as well as experiences of separation from children and plans for reunion with children. Integral to their reintegration, we also examined how Black fathers maintained their identities as fathers and connections with their families despite the stress of incarceration, using a risk and resilience framework and an ecological perspective. We focused on jail incarceration because, although it is the most common form of incarceration in the U.S., recidivism from jail (versus prison) is understudied, especially in the family context.

### 1.1. Incarceration in Jails

Year-end population statistics published by the U.S. Bureau of Justice Statistics indicate that in 2019, 1,430,800 people were housed in state or federal prisons and 734,500 were incarcerated in jails across the U.S. [1]. Jails are local corrections facilities usually run by sheriff’s departments or city governments; they house those detained, awaiting conviction or sentencing, or serving sentences for misdemeanor crimes, usually for under a year. In contrast, prisons are state or federal facilities that house those convicted of felonies, typically for more than a year. Year-end or daily population statistics, however, do not tell the whole story regarding mass incarceration in the U.S. [3]. Yearly statistics are also an important part of the mass incarceration story. More than 10 million admissions to jails occur each year across the country, including many individuals who are released and reincarcerated within the same year [7,8].

Within jails, most people are awaiting conviction or sentencing or unable to post bail. Indicators of the systemic racism inherent in the courts include Black individuals being more likely to experience pretrial detention and having higher bail limits set than White people [4,9]. About 170,000 people on any given day are serving sentences for conviction of misdemeanor crimes [2]. These are usually low-level crimes such as disorderly conduct, vandalism, trespassing, petty theft, prostitution, public intoxication, simple assault, reckless driving, discharging a firearm, or possession of cannabis, nonpayment of child support, or bail jumping, depending on the jurisdiction. Another substantial reason for jail incarceration is revocation based on violation of the technical terms of parole or probation. At least one in four people who go to jail are rearrested within the same year, with many of those who return affected by mental illness, substance abuse, and poverty [3,10] Although mental illness and substance abuse are even more common in jail than in prison (e.g., [10,11]), a recent paper found that Black parents in jail were less likely to show mental illness than White parents in jail [12], suggesting different pathways to incarceration. It is unknown, however, if there are unique pathways to recidivism (and reintegration) among Black incarcerated fathers.

Mass incarceration, whether in jail or prison, is a form of systemic oppression in the United States with well-documented and profound impacts on Black families [13]. Incarceration not only takes a family member—usually the father—out of the provider role, which can lead to family financial instability and material hardship, but also burdens families with additional costs such as legal fees, court fees, cash bail, and costs for visits and phone calls [4,14]. Incarceration also decreases future economic mobility [15,16]. Moreover, often technical violations of the strict rules of probation and parole can lead to reincarceration within a short period of time, especially for Black men (e.g., [17]). In addition to these issues, many Black men, and women, most of whom are parents, are incarcerated in jail for relatively brief periods for crimes of poverty—unpaid parking tickets, nonpayment of court-ordered child support, unpaid debt or fines, or lack of the ability to pay cash bail [3,18]. Yet even short periods of incarceration can negatively affect one’s ability to pay rent or a mortgage or meet family and state obligations like the payment of child support. More arrests and incarcerations can feed into a vicious cycle as it relates to employment, family relations, and poverty. Yet amidst these systems of oppression, many Black families show enormous resilience and willingness to support incarcerated individuals [14,19].

### 1.2. Reentry and Recidivism

In 2019 alone, state and federal correctional facilities released 608,000 individuals [20] and 878,900 people were on parole, or the conditional release of an individual into the community after incarceration while still under correctional supervision [1]. About two-thirds of individuals released from prisons are rearrested within three years, with a 44.1% rearrest rate occurring within the first year after release [21]. Although there are fewer studies of jail recidivism, one study measuring jail reincarceration within the year following release from jail found a rate of 36.7% [22]. Recidivism rates among Black men are higher than any other ethnic group, representing structural discrimination and a depletion of resources available to families and children (e.g., [23,24]).

According to the Bureau of Justice Statistics [25], recidivism measures require three characteristics: (1) a starting event, such as a release from prison or jail, (2) a measure of failure following the starting event, such as a subsequent arrest, conviction, or return to prison or jail, and (3) an observation or follow-up period that generally extends from the date of the starting event to a predefined end date (e.g., 6 months, 1 year, 3 years, etc.). Some scholars use reincarceration in prison or jail as the measure of recidivism, whereas others use new convictions or arrests or some combination of the above [25]. In this study, we use new convictions within 1 year of release from jail because this information was readily available using state consolidated records systems.

Although being a parent is not consistently related to rearrest and reoffending, children often provide motivation for parents to succeed during incarceration and reentry [4,26,27]. Previous research has found that individual predictors of recidivism include age, race, gender, marital status, educational attainment, number of prior convictions, and employment [28,29], so these are important factors to consider in studies of recidivism and reintegration. However, child and family factors are important for incarcerated parents during incarceration and reentry as well, including parent–child contact during incarceration and family support [30]. In terms of methodological approaches, several studies have used an integrated mixed methods design which supports the use of qualitative and quantitative methods for the purpose of hypothesis testing and hypothesis generating on reentry for youth and adults [31,32] such as the method that we are utilizing in this study.

### 1.3. Child and Family Factors

In addition to its relation to the literature on incarcerated fathers, the present study may also be relevant for the wider literature on Black fathers. The social construction of Black fatherhood has progressed through various phases over the last two centuries of American social history. Evidence of responsible Black fatherhood behavior is well documented during the time of chattel slavery [33,34]. Furthermore, despite recurring barriers post-slavery which included the lingering effects of historical institutionalized and legalized racism, Black fathers were engaged in adaptive ways to serve as an active parent to their children [35,36]. More recent research on fathers and families consistently reveals that most Black males want to be present and involved fathers. In fact, the most recent literature highlights that role flexibility is most relevant for Black biological and social fathers, including the establishment of setting firm guidelines in the context of “a close, warm, and nurturing father–child relationship” [37,38,39].

A growing number of scholars who have examined how incarcerated individuals fare during reentry from prison have found that family support is critically important (see [19,40]). One such investigation is the Returning Home study conducted from 2001 to 2006 by the Urban Institute to document incarcerated individuals’ reentry following release from prisons in several states. In an analysis of Returning Home data, the researchers found that 80% of those who reentered the community from prison felt that family was an important factor in helping them to stay out of prison [41]. Family members provided multiple supports, including emotional, instrumental, housing, and financial support, and they often helped the formerly incarcerated individuals find employment [41]. In addition, fathers who were more engaged in their children’s lives 3 months into the reentry period also were less likely to recidivate or violate the terms of their parole [42]. They also reported fewer depressive symptoms and worked more hours per week than fathers who were less engaged with their children, suggesting better integration into the community and family. According to the Centers for Disease Control and Prevention, Black fathers are the most engaged with their children across a range of involvement activities compared to U.S. American fathers of other races [9]. A key predictor of engagement in children’s lives among incarcerated Black fathers was how much contact the father had with the child during the prison stay [42].

Another more recent study that focused on returning fathers is the Multi-site Family Study on Incarceration, Parenting, and Partnering, which was conducted as part of federal demonstration programs focusing on incarcerated fathers’ family involvement. Nearly 1500 fathers and their female partners were followed longitudinally. The researchers found family support was critical in helping fathers adjust in the community, although post-release supervision practices, and policies did not support incarcerated fathers’ families [30]. Again, father–child contact during incarceration was important in helping fathers adjust to family life following reentry. In an analysis of a subset of fathers who provided interviews, researchers found that fathers were more likely to live with and financially support their children upon release when children were younger, there was more father–child contact during incarceration, and when the father–mother relationship was more positive [43].

Investigators found that among the individuals reentering their communities in the Boston area following a prison incarceration, family of origin support was important for success [19]. Western’s study, although not specific to incarcerated parents but included many incarcerated parents, indicated that female relatives such as mothers and sisters were particularly supportive during reentry. For the reentering parents in the study, caregivers of children did not play a significant supportive role but rather served as gatekeepers of parental contact with children. The role of fathers’ relationships with the caregivers of their children—typically children’s mothers or grandparents—remains to be seen regarding reentry success.

In addition to these large studies, smaller studies have also investigated paternal experiences of reunion with their children. For example, in a qualitative study of 19 fathers who had at least monthly contact with their children and who had been released from prison in the past year, investigators found that fathers felt deeply committed to their children even though they experienced multiple reentry challenges associated with poverty and inequality during reentry [26]. In another qualitative study researchers interviewed 10 African American fathers reentering the community following a prison stay and found themes related to unaddressed childhood trauma, low self-esteem and self-worth, and family reunification [44]. All the fathers discussed reuniting or enhancing their relationships with their older children because “those were the relationships they felt needed the most work” [44] (p. 249). However, barriers often existed regarding their access to younger children, especially conflict with the child’s caregiver, who regulated access to the child.

Only a few studies have been conducted on recidivism or reentry when a father is in jail (rather than prison), despite the pervasiveness of jail incarceration. In one exception, the Vera Institute of Justice conducted the Close to Home Project examining incarceration and reentry in relation to family factors for three jails [45]. They found that the family relationships of jailed individuals, 67% of whom were parents, were important. Most individuals incarcerated in jails, similar to those in prisons, relied on family support during their incarceration and during reentry into the community [4,45]. The limited research focusing on reentry for parents incarcerated in jails means that it is unclear whether or how children’s caregivers or other family members maintain family connections during incarceration, including father–child contact, or if this is predictive of recidivating or not recidivating following release from jail.

### 1.4. Theoretical Framework

We apply a risk and resilience framework [46] that examines risks and protective factors related to positive outcomes for individuals living with significant risk within the ecology of the family and other social environments in which human beings develop and interact. Resilience in Black men, and specifically for low-income Black fathers following incarceration, is a largely understudied phenomenon, even though incarceration remains a major risk for low-income Black men. In fact, the ongoing inequities, segregation, and discrimination that are at the core of the history of Black people in the United States continue to inform institutions and structures that are vital to life and mobility. Informed by these conditions, incarceration is an existential risk for low-income Black men [47]. Understanding the factors associated with positive outcomes (e.g., low recidivism or successful reentry) for formerly incarcerated fathers, is an important consideration for improving how U.S. American systems of justice interact with this population, and how children and families can be supported through this experience.

While the literature on paternal incarceration explores how fathers’ incarceration disrupts and negatively affects the family, and specifically children through financial hardship, loss of emotional support, and loss of a parent, support from the family may also have effects on Black fathers who are incarcerated [14]. The risk and resilience framework proposes that assets (factors internal to the individual such as motivation to engage with the child post-incarceration) and resources (factors external to the individual, like family support) play critical roles in lessening, and sometimes nullifying, the effects of risk exposure [46,48] such as incarceration and negative institutional contact. We conceptualize family support as a resource (external to the individual) for Black incarcerated fathers. Moreover, we expect that resources like quality of the coparenting or caregiving relationship and family support could enhance father motivation and intention to engage with their children (asset or internal cognitive factor). This interplay of assets and resources in the face of significant risk may reveal important associations for decreasing recidivism.

Family support is a resource that enriches resilience in Black men who were formerly incarcerated. One key component of resilience in this population is the creation of one’s own supportive environment [49]. Immediate family and close friends are often the core of these support networks, which improve the odds of positive outcomes and provide resources for managing the stress of living in a racially discriminatory and unfair system that limits access to success or mobility [50]. In fact, in studies of Black men’s resilience, participants often identify stressors and challenges like incarceration, racial microaggressions, employment difficulties, and navigating their difficult neighborhood conditions as the main risks to positive outcomes. However, they identify internal assets like perseverance, self-reflection, and a determination to overcome as contributing to their overall resilience in the face of these risks [49,51]. Black men also identify religion/spirituality and support from key social networks like family and friendships as pivotal to their resilience [49,50,52].

The criminal justice system has historically been unjust and unequal in its treatment of Black men, where in the United States they are 6.2 times more likely to be incarcerated compared to White men [47]. Black incarcerated fathers, however, also exist in the social ecology of their families. Appending a socio-ecological framework [53] to this study allows for an appreciation of the intersecting challenges (e.g., incarceration and unemployment) that Black men face within their communities while also centering the critical roles that social resources like family support play in improving the odds of success for Black men in low-income contexts. This ecological perspective of resilience accounts for the traditional links between the individual and environmental risks, but also brings to focus the links between the individual, community, and other social levels [50,54,55].

Research suggests African American fathers are often met with structural and institutional barriers that inhibit their opportunity to financially support their children [56]. Poor fathers will often transform their ascribed role as the breadwinner into a more accessible and achievable role such as being a caretaker of their child(ren). It has been suggested that a more fluent and inclusive term is needed to capture the essence of the fathering role [57] as these roles of caretaker or breadwinner are further compromised by the “shock” of an incarceration. Unfortunately, in general Black men are rarely studied as parents and when they are, it is often as absent fathers. This research illuminates the need for additional research that documents the fathering experience of incarcerated Black men in jail.

Without highlighting the well-documented structural disadvantages of Black male identity in the United States, and without accounting for the historical and enduring inequities in how U.S. American systems of justice police the bodies of Black men, we would miss a major element of the story of Black fathers who are incarcerated. Against this narrative backdrop, we focus on the stories of incarceration and child nurturing told from the perspectives of Black fathers. Recognizing how incarceration and other justice system experiences create stratifications within the Black community [58,59] we focus on fathers’ employment as a key resilience factor in understanding the impact of incarceration on Black fathers. A Bureau of Justice Statistics report indicates that the rate of imprisonment among Black Americans has dropped by 34% since 2006 [20], though young and less formally educated Black males are at greatest risk of incarceration, and thus at the lower end of the social strata in Black communities. Our study therefore features the stories and experiences of Black fathers who are incarcerated, and centers on their resilience through a socio-ecological focus incorporating resources and assets related to their post-incarceration outcomes.

### 1.5. The Current Study

In the present longitudinal mixed methods convergent study, we focus on Black fathers who were incarcerated in jail during the initial data collection. We enrolled fathers with young children for three reasons. First, most experiences of parental incarceration occur before children are 9 years of age [60]. Second, researchers found that when children were younger, fathers had an increased chance of living with and financially supporting their children following paternal release from prison [30,43]. Third, fathers in the criminal justice system who have young children are understudied, especially during reentry [27]. Thus, in the present study we include fathers who had at least one child between 2 and 6.5 years of age at the time of the initial data collection. Additionally, one of their children was randomly selected for participation in the study (referred to as the “focal child”). We focus on Black fathers in jail because of racial disparities in the criminal justice system, high recidivism rates, and because jail is the most common—albeit understudied—form of incarceration in the U.S., thus filling a gap in the literature. We include a qualitative component to the study to further explore fathers’ experiences of separation from children during incarceration, their identities as fathers, and plans for reunification with children to expand on factors that occur during incarceration that can be important during the reintegration process.

### 1.6. Research Questions

The study addresses the following questions:

1. How do Black fathers experience fatherhood in the context of incarceration and does this relate to their plans for reunion with their children following release?
2. What is the rate of new convictions and incarcerations for this sample of Black fathers with young children 1 year after the father’s reentry from jail?
3. Does frequency of father–child contact mediate the relation between father–caregiver relationships and not recidivating and is this indirect effect conditional on other factors?

We examined child-related variables (father planned on living with the child and father–child visits and phone calls during incarceration); family variables (family support and father–caregiver relationship quality), individual variables (paternal age, education, pre-incarceration income, pre-incarceration employment, mental health problems, and alcohol and drug use), and system-related variables (current incarceration related to nonpayment of child support or revocation). In our quantitative analysis, we hypothesized that father–caregiver relationship quality and family support would interact in predicting frequency of father–child contact during incarceration. We also hypothesized that frequency of father–child contact would mediate the relation between father–caregiver relationships and not recidivating, controlling for demographic variables, pre-incarceration employment, mental health/substance problems, and whether or not the father was incarcerated for not paying child support. We expected the indirect (mediated) effect to be conditional on whether or not the father planned to live with the child following release, with stronger associations expected when fathers planned to live with their child.

## 2. Methods

### 2.1. Research Design

This study is a longitudinal mixed methods study that utilizes a single-phase or convergent mixed method design involving the separate collection and analysis of quantitative and qualitative data for us to best understand the phenomena. Utilizing this design for the research provides us with the opportunity to merge the two data sources by bringing the separate results together for ease of interpretation and facilitation of a more integrated analysis [61]. It was our intent to relate the quantitative results to the qualitative findings, which reflected the strengths of our research team.

### 2.2. Sample

The sample was drawn from a larger multi-method, multi-respondent study of incarcerated parents with children between 2 and 6.5 years of age (mean = 4.1 years, SD = 1.3; (see [62,63]). From the larger study, 86 fathers were Black or biracial, with biracial fathers identifying as Black and one other race. Two of the fathers were still incarcerated for the same conviction as when they were initially assessed in jail. They were excluded from this report. The remaining 84 fathers ranged in age from 18 to 46, with a mean of 29.3 years (SD = 6.3). Their education ranged from 9 to 16 years, with an average of 12.3 years (SD = 1.4). Most fathers reported that they had never been married (78.6%), whereas others were married (16.7%) or divorced (4.8%). They had served an average of 45.7 days in jail (2–210 days, SD = 46.3), with primary and secondary offenses listed in Table S1 in Supplementary Materials. The most common offense was a revocation for a technical violation of the terms of parole or probation, and the second most common offense was nonpayment of child support. In addition, less than 1 in 10 of fathers in the sample was incarcerated for a first offense, with the number of prior arrests ranging from 0 to 52, with a mean of 8.9. Pre-incarceration annual self-reported income ranged from $0 to $115,200, with a mean of $13,669 (SD = $17,875), median of $9564, and mode of $0. Forty-three of the focal children were girls; 64.3% of fathers lived with their children prior to incarceration and 64.3% of fathers planned to reunite with their children following incarceration (although these proportions overlapped, they did not represent the same fathers). In addition, 72.6% of fathers had retained legal custody of the focal child, and none of the fathers had their parental rights terminated.

### 2.3. Procedure

Recruitment efforts began with the jailed parent. Weekly, jail administrative staff provided either the names of newly sentenced parents who had children between 2 and 6 years of age or access to a database with this information. We identified incarcerated parents who then participated in a brief initial screening with a trained researcher to determine if they met research criteria indicating that they: (1) were at least 18 years old, (2) had a child who lived with kin within the county in which the incarcerated person was serving time (or an adjacent county), (3) had retained legal rights to the child and had not committed a crime against the child, (4) had cared for the child at least part of the time prior to incarceration, (5) could understand and read English, and (6) had already been sentenced to serve jail time or were charged with committing a misdemeanor crime that would result in jail (rather than prison) time. For this analysis, we focused on Black fathers.

If the incarcerated parent had more than one child in the age range, one child was randomly selected for participation (referred to as the “focal child”). Incarcerated parents who met criteria were invited to participate in the study, and those who agreed signed informed consent forms and participated in an interview and self-administered questionnaires. For about half of the families, children and children’s caregivers also participated in the study [62]. At initial enrollment and at 1 year following release, we examined public court records in the state in which data were collected to determine the initial offense and subsequent recidivism. The study was approved by the Institutional Review Board from our university and a National Institutes of Health Certificate of Confidentiality was used. We were unable to compensate incarcerated parents due to jail policies.

Two jails were sources of enrollment for this sample, both of which were run by county sheriff’s departments. The first jail from which we recruited fathers (*n* = 41) is in a large urban community (823-bed capacity, 8000 annual admissions, 788 daily population, and 79% men). Other incarcerated fathers (*n* = 43) were enrolled from a second jail in an urban community that holds a mix of individuals from urban and rural locations (876-bed capacity, 13,000 annual admissions, 800 daily population, and 84% men). Both jails disproportionately incarcerated Black individuals relative to the state’s racial demographics. These characteristics are similar to other jails in the Midwestern region of the United States.

### 2.4. Measures

Descriptive statistics for each measure are shown in Table 1.

#### 2.4.1. Demographic and Family Information

Based on pre-study interviews with incarcerated fathers, we collected self-reported information about paternal age, race, education, number and ages of children, pre-incarceration income, hours of work per week, current offense, length of incarceration, prior arrests and incarcerations, and previous mental health and substance abuse problems. Most fathers (93%) had been incarcerated previously, most often for short stays in jail, although 18 (21%) fathers had served 18 months or more in prison. For those who had a sentence while in jail, sentences ranged from 5 days to 1800 days. This range includes the original sentences for the 24 fathers in jail on a revocation. Twenty fathers in the study were awaiting sentencing. Fathers had been separated from their children because of this incarceration between 1.5 and 450 days, with a mean of 70 days (median = 32 days; mode = 30 days). Excluding fathers who were in jail on a revocation, the modal length of separation was 14 days. The focal children ranged from 1 year to 6.5 years at separation from fathers (M = 3.6, SD =1.4).

We asked fathers if they had lived with the focal child right before their current incarceration and if they planned to live with that child following release. In addition, we asked fathers multiple open-ended questions, including: What was the separation from your child like for you at first? How are you doing with it now? What was the separation like for your child, and how is your child doing now? What is the most difficult part about separation from your child? Do you receive visits from your child? If so, how often? Who brings the child? If not, why not? Do you talk on the phone with your child? If so, how often? What are your plans for reuniting with your child? Responses were written down as close to verbatim as possible because the jails did not allow audio recording of interviews. Interviewers used prompts if the respondent did not initially answer the question. Responses were analyzed using a grounded theory approach (see Plan of Analysis section).

Fathers also shared information about their pre-incarceration employment; 50 (59.5%) fathers were employed prior to incarceration, with 35 being employed at least full-time or even working more than one job, and 15 having part-time work. Jobs ranged from administrative (*n* = 2) or clerical/sales work (*n* = 2) to skilled manual employment (*n* = 12) to machine operation (*n* = 13) or unskilled work (*n* = 20). One father did not report his occupation.

#### 2.4.2. Recidivism

We used the Circuit Court Access system that contains public records of criminal convictions and incarcerations to look up individuals at the time of study enrollment to determine their offense and 1 year following their release from jail. We used new convictions as the measure of recidivism (coded as recidivating = 0; not recidivating = 1). We recorded new incarcerations and community supervision.

#### 2.4.3. Father–Child Contact

Frequency of father–child contact was coded from interview questions during the fathers’ time in jail. We asked how often the child visited in one question and how often the incarcerated father talked to his child on the phone in another question. Some fathers provided a weekly average. However, some fathers said 1 time per month (which we calculated into a weekly average) or “1 time since I was incarcerated” (which we calculated into a weekly average based on the date of incarceration and the date of the interview). We combined the information from visits and calls into a total contact score, as they were significantly correlated, *r* = 0.314, *p* = 0.004. We did not include written correspondence due to the young age of focal children. Weekly averages for phone calls with the focal child ranged from 0 to 7 (M = 1.82, SD = 2.57). Weekly averages for visits ranged from 0 to 3 (M = 0.32, SD = 0.58). About 60% of fathers did not receive visits from the focal child; for some fathers, this was their choice, but for others, it was because of factors outside of their control (e.g., the caregiver refused to bring the child or the availability and cost of transportation were barriers). Approximately 42% of fathers did not talk with the focal child on the phone. However, the types of contact were compensatory to some degree, with 72% of fathers receiving visits or phone calls with the child. Total contact ranged from 0 to 9 calls/visits per week (M = 2.13, SD = 2.81). Due to the somewhat skewed distribution of the total contact variable, we used a square root transformation in analyses.

For the 34 focal children who visited their fathers in the jail, most (79%) were accompanied by their mothers. About 20% of children were brought by grandparents, aunts, uncles, or other extended family members (usually paternal), and less than 1% were brought by the father’s new partner.

#### 2.4.4. Family Support

To evaluate family support, we used the family subscale of the Multidimensional Scale of Perceived Social Support (MSPSS) [64]. Statements referring to the family are rated on a 1 to 7 scale, with higher numbers indicating more agreement with the statement. The four statements about the family include “My family really tries to help me”, “I get the emotional help and support I need from my family”, “I can talk about my problems with my family”, and “My family is willing to help me make decisions”. Respondents were also asked to list their relation to the family member(s) who fulfilled the type of support assessed. Fathers’ scores ranged from 4 to 28 (M = 20, SD = 7) on the family subscale, and they most often listed their mothers as supportive family members, although listing multiple family members was also common. The MSPSS has good psychometric properties [65]. Cronbach’s alpha for the family subscale was 0.90 in this sample.

#### 2.4.5. Fathers’ Feelings about Children’s Caregivers

The Inventory of Family Feelings (IFF) [66] was used to assess fathers’ perceptions of relationships with their children’s caregivers. The IFF is a 38-item self-report measure of interpersonal affect that shows patterns of conflicted relationships and warmth or closeness in dyadic family relationships. The IFF has high reliability, good construct, and concurrent validity, and has been used to index quality of relationships between spouses, parents and children, and other family members (e.g., [67]). Scores range from 0 to 38, with higher scores indicating more positive affect, including warmth, loyalty, trust, and respect, toward family members and lower scores reflecting conflicted relationships. The IFF was chosen for this study because it has been used with incarcerated parents previously [62,68], and it is one of few measures that applies to a variety of family relationships (e.g., parent–parent, parent–grandparent, and parent–other relative). In this sample, 89% of focal children were living with their mothers, but 11% lived with a grandparent or aunt or uncle (usually maternal). Fathers’ IFF scores regarding their children’s caregivers (IFF-CG) ranged from 0 to 38 (M = 24, SD = 13). Cronbach’s alpha for the IFF-CG in the present study was 0.89. 

#### 2.4.6. Alcohol and Substance Use

The Michigan Alcoholism Screening Test (MAST) was used to assess incarcerated fathers’ self-reported alcohol abuse in the previous 12 months [69]. Twenty-five items comprise the measure. Scores range from 0 to 53, with higher scores indicating higher risk of alcohol abuse. In the current study, the MAST had a Cronbach’s alpha of 0.89. The Drug Abuse Screening Test (DAST) was used to assess incarcerated fathers’ self-reported drug abuse in the previous 12 months [70]. Twenty items comprise the DAST. Scores range from 0 to 20, with higher scores indicating more drug abuse severity. In the current study, the DAST Cronbach’s alpha was 0.93. On the MAST, 44 fathers were characterized as having no alcohol problems, whereas 8 showed borderline problems, and 32 showed evidence of alcohol problems. On the DAST, 39 fathers were characterized as not having a drug problem, whereas 45 showed evidence of drug problems.

### 2.5. Plan of Analysis

#### 2.5.1. Qualitative Analysis

We analyzed the open-ended responses using a grounded theory approach. As this was a secondary data analysis, the interview questions did not change throughout the process, as they would if using traditional grounded theory methodology [71]. The analysis was completed using NVivo 12 (QSR International 2022, Burlington, MA, USA). (Two authors of the study were the primary analyzers of qualitative data—both white middle class women, one a mother; they reviewed the transcripts and created memos to create open codes, met weekly to review the memos and open codes and decide which could be combined, entered the data into NVivo based on words and open codes that were noted in the memos, and discussed the open and higher order codes to reach agreement; to get multiple rounds of feedback, they also discussed the memos and higher order codes in detail with other study authors, both Black middle class men, one a father).

We began by creating open codes (as defined in grounded theory methods) based on memos created after reading fathers’ responses to the questions: (1) How did you adjust to the separation at first? (2) How are you adjusting to the separation now? (3) How did your child adjust to the separation at first? (4) How is your child adjusting to the separation now? and (5) What are the most difficult parts about being separated from your child? These responses elicited 55 open codes. As these codes were analyzed and higher order codes and themes began to emerge, we brought in responses to an additional eight questions to continue the analysis. These questions are: (6) Who is your child with now? (7) Was this your choice? (8) Are you satisfied with the arrangement? (9) How did you decide on this person? (10) If your child does not visit you while you are incarcerated, what are the reasons? (11) What concerns do you have about your child? (12) What are your child’s strengths? and (13) What plans do you have pertaining to your child upon release? With the introduction of these responses, we recoded all responses using the previously established open codes as well as producing more open codes. This process generated an additional 25 codes, for a total of 80 open codes. During this initial coding process, we kept detailed memos regarding the interactions, connections, and outliers among the codes [72,73].

Once the open codes were established, we continued with the grounded theory method of organizing and arranging the codes into selective and eventually theoretical codes. This process involved additional memoing and visual concept mapping. The memoing process, or running notes, allowed us to create an iterative record of our thoughts while engaged in active coding, including documenting ideas, feelings, and a rationale for the coding. We also used the memos as the basis for discussion among study team members.

The process for dealing with discrepancies and reaching consensus during the feedback/review sessions was initially done by the primary analyzers as explained in the previous paragraphs. The primary analyzers reviewed the memos and held discussions regarding the patterns that were emerging for theory building. When disagreements occurred, it provided an opportunity to rereview memos and create summary memos and reach consensus on the concepts. Subsequently, two additional researchers engaged in the process when dealing with a few lingering discrepancies to reach consensus during our feedback and review sessions.

#### 2.5.2. Quantitative Analysis

**Figure 1:**
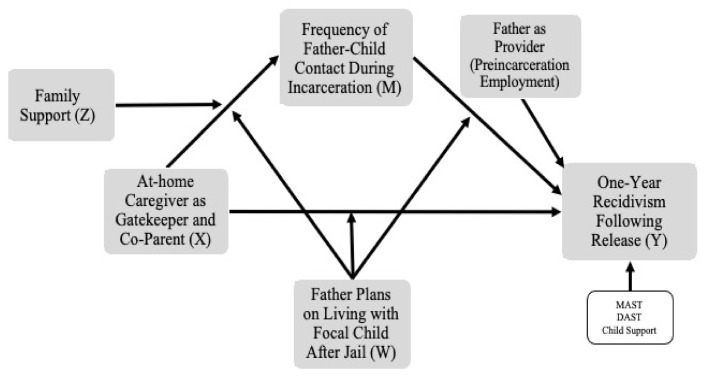
Moderated Mediation Model Tested in the PROCESS Macro for SPSS.

We began by reporting descriptive statistics about fathers’ recidivism. Subsequently, we evaluated a moderated mediation model (Figure 1) using Hayes’ PROCESS macro v3.5 [74], model number 61, executed in SPSS v.26 (IBM Corp, Armonk, NY, USA). [75]. We evaluated the indirect path from father–caregiver relationship quality (X) to the outcome, recidivism (Y), via the mechanism of contact with the focal child (M). We also examined two moderators: paternal plans to coreside with the child following incarceration (W) and family support (Z). The PROCESS macro mean centers continuous variables used in the construction of interaction terms and generates 10,000 bootstrapped samples to calculate 95% bias-corrected confidence intervals; interactions significant below the *p* < 0.10 level are examined at multiple levels of the moderator and tested for significance. The PROCESS model also estimates regression coefficients of all direct and indirect paths using ordinary least squares regression for continuous outcomes (M) and binary logistic regression for the recidivism outcome, also using 10,000 bootstrapped samples to calculate 95% bias-corrected confidence intervals.

The model also examined effects of paternal pre-incarceration employment in relation to recidivism. We controlled for alcohol problems (MAST category), drug problems (DAST category), and whether the current incarceration was for nonpayment of child support. We selected these covariates because they were theoretically related to and were correlated with the mediator or the outcome. We assessed additional covariates (e.g., father’s age, education, and income; number of prior arrests; pre-incarceration coresidence with the child; paternal satisfaction with the caregiving situation; if current incarceration was a revocation; history of mental health difficulties; and “father as protector” coded from the qualitative analyses) but rejected them because they did not relate to the outcome or mediator, or they were too highly correlated with other similar predictors. Reported effects were characterized as small (*r* = 0.10), moderate (*r* = 0.30), or large (*r* = 0.50) using Cohen’s benchmarks [76]).

Power analyses for the multiple regression and logistic regression analyses, conducted using G-Power 3.1 [77,78], are reported below.

## 3. Results

### 3.1. How Do Black Fathers Experience Fatherhood in the Context of Incarceration and Does This Relate to Their Plans for Reunion with Their Children following Release?

The findings of the qualitative analysis that address the first research question are organized into three themes that demonstrate the ways in which fathers experienced fatherhood, while simultaneously experiencing separation due to incarceration. These themes represent three distinct fatherhood identities: father as nurturer, father as protector, and father as provider. Throughout the results section, pseudonyms are used in place of participant names to protect confidentiality.

#### 3.1.1. Father as Nurturer

Throughout the interviews, the most frequently referenced fatherhood identity was father as nurturer. This theme broadly encompasses the open code, “to be there for…” Many of the responses reflected the idea of simply wanting “to be there for [the child, the family, the special experiences, the routine moments]”. This includes simple aspects of day-to-day life, such as hearing their children talk to them. For example, one father said that he felt particularly connected to his daughter “when she says Daddy” and that he missed this simple statement during incarceration. Hearing his young daughter call him “Daddy” made him smile and feel positively about his role as a father, and he looked forward to being able to experience such interactions following release from jail. Anticipating such interactions with his daughter also helped provide motivation to stay out of jail. From this selective subcode, we see one of the primary ways that fathers experienced their physical separation from their children. This subcode reflects the importance that fathers placed on simply being present in their children’s lives. One father, Kaenan, summed this up succinctly when discussing the most difficult part of the separation: “Not waking up seeing her every day. Not seeing her get ready for daycare every day. Putting her hair in a little ponytail”. From these responses we see the value that fathers assign to their ordinary day-to-day interactions with their children. Routine interactions were cited throughout the interviews as the experiences fathers missed most during incarceration and as what they were most looking forward to engaging in post-release. Another father concisely explained the importance of this nurturing connection when discussing his release plans: “Just being a dad, waking up and seeing [my son] every day”.

#### 3.1.2. Father as Protector

The second distinct fatherhood identity that emerged from the interviews was the idea of father as protector. This identity was highlighted in two ways throughout the interviews. The first was aligned with a traditional understanding of the ways in which fathers act as emotional security and physical protection from potential dangers. This idea was most prominent when fathers referred to children’s living arrangements during paternal incarceration, including wanting to protect children from harm or mistreatment.

Within this identity we see the ways in which the justice system interacts with a father’s perception of their role within their child’s life. When fathers were dissatisfied with the child’s current caregiving situation, they expressed acute concerns regarding their child’s safety and well-being. For example, reflecting on his concerns for his daughter, one father, Darius, responded, “Who she’s around, the company her mom keeping. I’m always worried about it. It’s my daughter. I’m mad I can’t be there for her”. When fathers expressed satisfaction with the child’s caregiver, concerns were more general and broadly encompassed children’s health, academic achievement, and happiness. For example, when asked about his concerns for his daughter during his incarceration, a different father, Levell, responded, “Just not being there as a parent. My only concerns are about me not being around. No general fears, just a lot of ‘what ifs.’ She’s got the best mom in the world, she’s so strong”. It is important to note that in many cases, the father’s view of the mother as a caregiver was separate from their partner relationship. For example, Levell, who is quoted above, reported lower than average responses for his relationship with the child’s mother; however, he still viewed her as a positive influence in his child’s life.

The second distinct demonstration of father as protector was through fathers protecting their children from the justice system and habituation towards carceral settings. Most commonly, fathers showed this protection by foregoing visits from their children during incarceration. The interviewees demonstrated a pervasive belief that interacting with their children in the jail may lead to their children becoming habituated to the carceral setting and therefore make them more susceptible to criminal justice involvement. One example of this pervasive sentiment is demonstrated by one father, George, when asked why he did not receive visits from his son. He responded by saying, “[I] Don’t want him to see me this way—I want him to be on a different path—I don’t want him to become comfortable with seeing me in jail”. Another father directly related his own experience of paternal incarceration to that of his son’s by saying, “I don’t want him here—when I was growing up, I saw my dad through the glass”. In addition to self-distancing, some fathers talked about the ways in which children’s caregivers acted as gatekeepers of father–child contact. This gatekeeping was primarily related to poor father–caregiver relationships, including no-contact orders. Additionally, some caregivers purposefully kept children from the incarcerated fathers in an attempt to protect them from both the criminal justice system and confusion of seeing their father incarcerated.

#### 3.1.3. Father as Provider

Throughout the interviews, many fathers discussed the importance of acting as the financial provider for their children and family; however, within the context of incarceration, many fathers were unable to continue as primary financial providers and were therefore stripped of that identity. Within the analysis, many of the fathers who spoke of their roles as providers did so in a way that highlighted their current inability to fill this role in their families. However, many fathers remained hopeful about resuming the role as provider in the future. For example, one father, James, said, “I’ll get released in a week, so that leaves me 20 days to get a job and a paycheck so I can get her a birthday present. If I can’t do that, it will be a real depressor”. In addition, many fathers showed a desire to be the provider through their plans with their families after release, such as plans to help their families become financially stable and plans to take their children on trips. We see this through one father’s, Rachard’s, description of his plans immediately following his release, “I was hoping to have enough money to take her to Disney World or some place. I’ve been gone a long time, so I’ll take her to do something fun”.

### 3.2. What Is the Rate of New Convictions and Incarcerations for This Sample of Black Fathers with Young Children 1 Year after the Father’s Reentry from Jail?

To address the second research question, we recorded new convictions and new incarcerations. In this sample, 36.9% of fathers had a new conviction within a year following release from jail, which is our measure of recidivism in this study. About 10.7% had a new incarceration related to the new conviction. Overall, 63.1% of fathers had no new conviction in the first year. In addition to the 36.9% with a new conviction, another 8.3% had an open criminal case.

### 3.3. Does Frequency of Father–Child Contact Mediate the Relation between Father–Caregiver Relationships and Not Recidivating and Is This Indirect Effect Conditional on Other Factors?

We conducted two analyses to answer the third research question: a multiple regression analysis examining predictors of father–child contact and a logistic regression analysis using new convictions as a measure of recidivism (coded as recidivating = 0; not recidivating = 1).

#### 3.3.1. Predictors of Contact with Children

The analysis predicting frequency of father–child contact during incarceration (M) was statistically significant, *R^2^* = 0.437, *F*(9,74) = 6.387, *p* < 0.001, with a moderate to large effect (Table 2a). Power for the overall effect size of 0.413, given the sample size and number of predictors assessed, was 0.994 for this analysis.

Fathers who planned to live with their child after incarceration (W) received more calls and visits during incarceration (M), *p* = 0.010. Moreover, there were three statistically significant interactions. The X*W interaction resulted in an *R^2^* change of 0.05, *F*(1,74) = 6.537, *p* = 0.013. The X*Z interaction resulted in an additional *R^2^* change of 0.06, *F*(1,74) = 7.590, *p* = 0.007, while the X*W*Z interaction resulted in an additional *R^2^* change of 0.13, *F*(2,74) = 8.301, *p* = 0.001.

**Figure 2:**
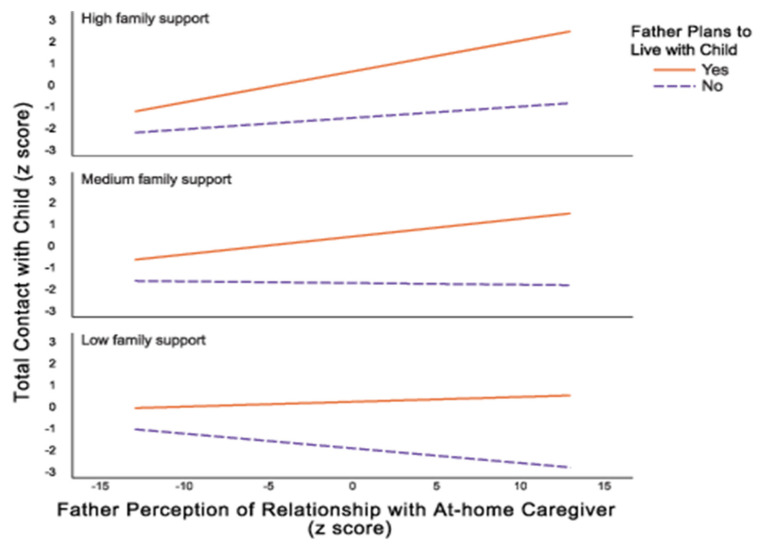
Three-Way Interaction of Family Support, Quality of The Father–Caregiver Relationship, and Paternal Plans to Live with the Focal Child on Father–Child Contact.

Tests of the X*W interaction revealed that when fathers planned to live with their child following release, positive father–caregiver relationships were associated with more father–child contact during incarceration, *p* = 0.001. However, when fathers did not plan to live with the focal child after incarceration, there was no association between father–caregiver relationships and father–child contact, *p* = 0.84. Tests of the X*Z and X*W*Z interactions indicated that when fathers planned to live with the focal child and received average or high levels of family support, positive father–caregiver relationships were associated with high levels of father–child contact, *p* < 0.01. When fathers did not plan to live with their children, father–caregiver relationship quality was unrelated to frequency of father–child contact, but—importantly—family support predicted contact frequency, *p* < 0.05 (Figure 2).

The only other significant predictor of father–child contact was child support; fathers who were incarcerated because of nonpayment of child support were less likely to have contact with the focal child during incarceration compared to fathers who were incarcerated for other reasons, *p* = 0.001. At the bivariate level, nonpayment of child support was unrelated to coresidence prior to incarceration or plans for coresidence following incarceration, however.

**Table 2:** (**a**). Predictors of Father–Child Contact Frequency During Incarceration: Multiple Regression (N = 84). (**b**). Predictors of Fathers Not Recidivating During the First Reentry Year: Logistic Regression (N = 84).

**(a)**						
				**95% CI**		
**Predictor**	***β***	***SE***	***t***	**LL**	**UL**	***p***
Constant	−0.734	0.396	−1.853	−1.523	0.055	0.068
IFF-CG (X)	−0.003	0.013	−0.248	−0.030	0.023	0.805
Plan to Live w/Child (W)	0.524	0.199	2.635	0.128	0.921	0.010
IFF-CG * Plan to Live w/Child (X*W)	0.040	0.016	2.557	0.009	0.072	0.013
MSPSS Family (Z)	0.006	0.013	0.439	−0.021	0.033	0.662
IFF-CG * MSPSS Family (X*Z)	0.003	0.001	2.755	0.001	0.005	0.007
Employed Prior to Incarceration	−0.090	0.188	−0.482	−0.464	0.284	0.632
Current Jail Stay: Child Support	−0.672	0.236	−2.841	−1.143	−0.201	0.006
MAST	0.064	0.101	0.636	−0.137	0.265	0.527
DAST	0.220	0.198	1.113	−0.174	0.614	0.270
Model Summary *R^2^* = 0.437, *F*(9,74) = 6.387, *p* < 0.001						
**(b)**						
				95% CI		
**Variables**	Coeff	*S.E*	OR	LL	UL	*p*
Constant	2.166	1.273	8.71	−0.329	4.662	0.089
IFF-CG (X)	−0.037	0.044	0.96	−0.122	0.049	0.400
Father–Child Contact (M)	0.702	0.831	2.02	−0.927	2.332	0.398
Plan to Live w/Child (W)	−1.243	0.716	0.29	−2.646	0.159	0.082
IFF-CG * Plan to Live w/Child (X*W)	0.115	0.889	1.12	−1.627	1.857	0.897
Father–Child Contact * Plan to Live w/Child (M*W)	0.006	0.052	1.01	0.910	−0.095	0.107
Employed Prior to Incarceration	1.109	0.532	3.03	0.067	2.151	0.037
Current Jail Stay: Child Support	−0.169	0.696	0.84	−1.533	1.194	0.808
MAST Total	−0.032	0.287	0.97	−0.595	0.531	0.912
DAST Total	−0.843	0.583	0.43	−1.985	0.299	0.148
Effect of M*W on the probability of Y = 1, Wald 𝟀^2^(1) = 3.56, *p* = 0.059						
Model Summary 𝟀^2^(9) = 15.790, *p* = 0.071 *R^2^*_McFadden_ = 0.143, *R^2^*_Cox-Snell_ = 0.171, *R^2^*_Nagelkirk_ = 0.234						

Note. OR = Odds Ratio; CI = Confidence Interval; LL = Lower Level; UL = Upper Level; IFF-CG = Inventory of Family Feelings-Father’s Ratings of Child’s Caregiver; MSPSS = Multidimensional Scale of Perceived Social Support; MAST = Michigan Alcoholism Screening Test; DAST = Drug Abuse Screening Test.

#### 3.3.2. Predictors of Not Recidivating

Although the logistic regression model predicting the probability of not recidivating (Y) was not statistically significant, *p* = 0.071, with the pseudo *R^2^* statistics showing a small to moderate effect (Table 2b), individual variables reached statistical significance. Power for the overall effect size of 0.180, given the sample size and number of predictors assessed, was 0.781 for this analysis.

Paternal pre-incarceration employment related to increased odds of not recidivating, *p* = 0.037. Fathers who were employed just prior to their current incarceration were less likely to experience recidivism during the first reentry year. Although the Wald 𝝌^2^ statistic of the interaction between whether the father planned to live with the child following incarceration and father–child contact only trended toward significance, *p* = 0.059, we explored the potential conditional effect to see if it is similar to past research and to inform future research. Exploratory tests of the interaction showed that when fathers planned to live with the focal child, more father–child contact was related to less recidivism. However, when fathers did not plan to live with the focal child, more father–child contact was unrelated to recidivism.

Qualitative and quantitative findings are integrated in Table 3. Four key findings were supported by both the qualitative and quantitative analyses, including: (1) *father as provider for his children and family*, (2) *father as nurturer of his child*, (3) *father as the child’s protector from the criminal justice system*, and (4) *the importance of father–child contact and family support*. Regarding the first theme, fathers indicated that they are motivated to work, provide for their families, and “treat” their children. Being employed prior to incarceration may make it easier for fathers to find a job following incarceration in jail, thus decreasing recidivism after their jail stay and allowing them to enact their motivation as providers. The second theme reveals that fathers want to care for and nurture their children and thus, one of the most difficult parts of incarceration is separation from their children. Fathers indicated that having children provides motivation for lower recidivism in the future. One way that fathers can care for and nurture their children during incarceration is to have contact with them through visits and phone calls, although quantitative results did not support a link between father–child contact and recidivism. The third theme reflects the common concern of many incarcerated fathers—they did not want to expose their children to the carceral environment because of fear of intergenerational patterns of incarceration. These fathers often sacrificed seeing their children during visits; however, many of the fathers who preferred that their children did not come to the jail were able to talk on the phone to their children regularly instead. The final theme reflects that incarcerated Black fathers are dependent on children’s caregivers and other family members to facilitate contact with children. Fathers’ ability to stay in touch with their young children during incarceration depended on the willingness of children’s caregivers and fathers’ extended family members to bring children to visit or facilitate (and often pay for) phone calls. When fathers planned to live with their children during reentry, positive relationships with caregivers and family members were important. When fathers did not plan to live with their children during reentry, support from extended family appeared to help fathers bypass the gatekeeping role of children’s caregivers, thus highlighting the critical role of family support networks.

## 4. Discussion

Mass incarceration in the United States has disproportionately impacted Black communities, as more than half of incarcerated men are fathers of minor children [6]. These over-representations within carceral settings demonstrate structural inequality within the justice system as well as a pervasive depletion of resources available to families and children within Black communities. However, despite oppression from the negative sequelae of incarceration, many Black fathers maintain engagement with their families throughout their detainment and reintegrate into family life upon release. In this study, we used a longitudinal mixed methods convergence approach to better understand the experiences of 84 Black fathers of young children. The purpose of the study was to qualitatively examine the ways that Black fathers expressed their fatherhood identity in the context of incarceration and to quantitatively assess rates and predictors of recidivism within the sample. Both qualitative and quantitative analyses were designed to inform our understanding of potential factors that may facilitate reintegration in Black fathers with young children. Our inquiry applied a risk and resilience ecological perspective to help contextualize the experience of fatherhood within incarceration and reentry.

As we stated previously, the role of fathering is a social construction and multi-faceted [79]. Several scholars have documented research on Black fathering as mirroring the existing literature on fathering in general. Whereas some scholars support the notion that for Black fathers, economic security is fundamental in their ability to become fully engaged fathers [80], recent scholarship on Black father identity and incarceration highlights how prison or jail can interrupt the paternal identity process and enhance maternal gatekeeping but potentially strengthen some family relationships [81,82,83,84]. Our research contributes to the limited research on incarcerated Black fathers and their paternal identity and involvement with their children.

A particularly compelling qualitative finding from this sample highlighted the importance of extended family support as a resource during Black fathers’ jail incarceration and its connection to father–child contact. Consistent with our theoretical model, moderate to high levels of family support helped fathers to remain engaged with their families during the incarceration period through visits and phone calls with children. Interaction analysis showed that when fathers planned to live with their child and received average or high levels of family support, positive father–caregiver relationships were associated with high levels of father–child contact (including visits and phone calls). Additionally, when fathers did not plan to live with their children, the relationship between the father and caregiver was unrelated to the frequency of father–child contact; however, family support still proved to be impactful in predicting contact frequency. These relationships may become even more complicated when fathers return to the community and the mothers of their children have repartnered or engage in gatekeeping access to children—relationship quality and coparenting thus are critically important [19]. These findings add to the work of many scholars who have demonstrated the importance of family support during incarceration and reentry [19,40], including reentry from jail [45], through documenting various pathways of support and inclusion of fathers during confinement.

Whereas these results demonstrate how extended family support is related to the frequency of father–child contact, quality of the father–caregiver relationship remained salient in terms of caregivers acting as the gatekeepers of young children’s contact with incarcerated fathers, which has been documented previously [85]. Numerous fathers talked about wanting to see their children during their incarceration but not being able to because the children’s caregivers, typically children’s mothers, would not bring them to the jail. Depending on the circumstances and family dynamics, these gatekeeping efforts could be circumvented by other family members, especially children’s grandparents.

Father–child contact during incarceration is important because it helps fathers to stay engaged in their family’s lives as well as retain their fatherhood identity. During interviews with fathers, the most prominently expressed identity was fathers as nurturers. Whereas historically mothers have been described as the primary source of care and comfort for children, our findings contribute to the developing narrative of Black fathers as co-nurturers (e.g., [86]) even within the oppressive context of incarceration [87]. As fathers in the study reflected on the most difficult parts of their incarceration, many of them referenced the ways in which they miss “being there” for their child and partaking in the simple day-to-day activities of their family, such as hearing children call them “Daddy” daily and feeling connected to their children. For many fathers, reengagement in these routine activities served as the primary motivation for successful reentry. Additionally, many fathers spoke of their visits with their children as a way to cope with the overwhelming loss of routine contact with them. During incarceration, visits and phone calls with children helped fathers retain their identity as nurturers and carry it forward as they rejoin the family, which is important when considering long-term successful reentry. Our quantitative findings indicated that frequency of father–child contact was not directly related to recidivism in the first reentry year. However, exploratory analyses of a statistical interaction indicated that when fathers planned to live with the focal child after release, more father–child contact during incarceration related to less recidivism. The results are partially consistent with previous research [30,42] which found that father–child contact during incarceration was a key predictor of paternal engagement in the child’s life as well as general adjustment after release from prison. In addition, the tie to Black incarcerated fathers’ description of themselves as nurturers of their children is novel.

In addition to fathers as nurturers, many fathers expressed their identities as protectors of their children. The positionality of being both a father and an incarcerated man was prominent within this identity in that many fathers felt it was their duty to protect their children from the justice system, even when this entailed the self-sacrifice of not being able to see them during their incarceration. The risk and resilience theory often refers to this modulating behavior as an asset managed by ones’ internal control to succeed. Our qualitative results showed that many fathers chose not to receive visits because they were concerned about intergenerational cycles of criminal justice involvement. Many fathers were concerned that even visiting the jail setting could habituate their children to carceral settings and therefore increase their likelihood of future criminal justice involvement. These qualitative findings were reinforced by our quantitative findings which showed that when fathers expressed their primary identity as a protector, they received less in-person visits, yet they still stayed in contact with their children through phone calls. This is a novel finding that contributes to the literature documenting the identity alterations experienced by Black fathers who are incarcerated.

The final theme that emerged in our findings involved fathers as financial providers for their families. Throughout the parenthood literature, many fathers strongly identify as the primary providers or “breadwinners” for their families (e.g., [88,89]). This is an identity that has been afforded to many fathers through a history of male-dominated workspaces and wage gaps between male and female workers [90]. Within our study, the fathers actually experienced adverse work situations through employment disruption due to incarceration, lack of employment opportunities due to prejudices of criminal records, and less work opportunity due to racial discrimination in the workplace [15]. These adverse conditions make it incredibly difficult for them to fulfill their desired role of father as provider. Our qualitative results bolstered these well-documented findings, as many of the fathers spoke about their desire to financially support their family’s day-to-day living expenses as well as special family trips and presents for their children. Our quantitative findings show that even in the context of this extreme financial adversity, these fathers demonstrate resilience. Paternal pre-incarceration employment had an inverse relationship to the likelihood of recidivism within the first year of reentry. Employment prior to incarceration may make it easier for fathers to find employment upon release, as it establishes a work history and experience as well as strengthening the provider role, which could facilitate lower recidivism through increasing financial well-being and community integration.

### 4.1. Limitations

One of the limitations of the study, in addition to the small sample size, limited power to detect small effect sizes, and lack of generalizability, was that we were not able to collect child data or father interview data at the 1-year follow-up. We only had access to paternal recidivism data, as measured by public records, at that time point (in the primary state of data collection and neighboring states, not nationwide). Future studies should examine the reentry process and the effects of parental recidivism on children at different ages and should make use of nationwide recidivism data. In addition, recidivism can be defined in many ways—in this study that involved a jail sample, we chose to use the indicator of new convictions as our measure of recidivism. Future research should consider using more than one indicator of recidivism and include data about crimeless revocations. In addition, we did not have access to call logs or visiting records at the jail so we were not able to confirm fathers’ reports of phone calls and visits with children. However, corrections facilities generally do not record children’s visits or calls, but rather record the adult call or visit with the adult who is supervising the child. In the future, it may be helpful to also include the reports of children’s caregivers when collecting data about child visits and calls. We were unable to audio record interviews in the jails and thus, it was difficult to record in-depth interview data with incarcerated fathers, as fathers’ responses needed to be recorded by hand. An additional limitation to the study is that there were no member checks incorporated in the analysis of the data because it was a secondary data analysis. Therefore, it was difficult to locate the informants for the purpose of member checking. Our limited access to administrative data and informant family members reduced our ability to conduct triangulation.

Future research should attempt to collaborate with jails or prisons that allow use of audio recording. Moreover, families are often complex, with multiple children and caregivers, and we only selected one focal child for data collection and analysis, which likely oversimplifies the multiple family factors at play.

### 4.2. Implications

The findings of this study have implications for criminal justice system programs and policies that affect Black fathers in jail and during reentry. We recommend offering parenting programs in jail, increasing family support during jail incarceration and reentry, and facilitating contact with children during incarceration. As corrections systems begin to recognize the importance of family factors during and following incarceration, an increasing number of parenting programs have been offered in prison settings [91]. Such programs can reaffirm the identity of incarcerated parents and help them maintain and build positive relationships with family members from a distance [89]. Some of the more rigorously implemented and evaluated programs have even shown effects on recidivism. For example, researchers found that incarcerated parents who participated in the Parenting Inside Out parenting program had 41% fewer arrests within a year after release than participants in the control group (services as usual) [92]. It is also important to go beyond indicators of recidivism and examine other factors that reflect reintegration into families and society following incarceration [4].

Despite these encouraging findings and the importance of understanding parental identity during jail stays, few family-based programs are offered to parents in jails [45]. As most incarceration in the United States occurs in jails and few services are available, there are numerous opportunities to increase provision of family-based jail programs and jail reentry support services. It is most helpful if the services are gender-responsive, for both incarcerated men and women, culturally appropriate, and are integrated with other services that attend to mental and physical health, substance use, employment, and housing issues. Integrating the services can help support multiple parental identities, such as “parent as nurturer” and “parent as provider”. Similar to parents returning from prison, parents reentering from jail often rely on family support to reintegrate into the community [45], and children often provide motivation for incarcerated fathers to succeed during reentry [26]. Strengthening family relations, including father–child, father–caregiver, and father–grandparent relationships, during incarceration may help adjustment and parental identity during incarceration and with transitions into the community, which can impact recidivism rates, reintegration success, and well-being of individuals, families, and communities.

Offering opportunities for supported parent–child contact during incarceration, including low-cost or free phone calls and in-person or remote visits, can bolster parental identity during incarceration and increase resilience in both parents and children [93]. Child-friendly visits—involving preparation for children and families, child-focused activities, ample physical contact, opportunities to share snacks and meals, freedom of movement, modified security procedures, and contact between visits—help children and their incarcerated parents meaningfully engage with each other and often lead to positive child outcomes in the context of parental incarceration and improved mental health in incarcerated parents [94]. In-home remote visiting can also be a positive way for children and incarcerated parents to connect with each other, especially when in-person visits are not available (e.g., during a worldwide pandemic) or when families do not want to bring children into a carceral environment, such as when a parent or caregiver is exercising their role as “protector” (see [95] for a summary). Justice professionals have identified the need for intensive cognitive behavioral case management to address risk, need, and responsivity factors such as homelessness, substance abuse, and the lack of medical insurance for those released from prison, to reduce recidivism [96]. Scholars suggest that clinicians engage in personal reflection on their views about incarceration and incarcerated individuals, recognize the important cultural context of kinship networks, explore conditions of self-worth, and above all appreciate the impact of systemic inequality and for-profit corrections on the lives of racially marginalized individuals [97].

## 5. Conclusions

Overall, the results of this study highlight the resilience of Black fathers and their families, despite the risks associated with incarceration, including the importance of family support during incarceration, children as motivation for not reoffending, how the justice system weighs the bidirectional influences of fathers and families.

## Figures and Tables

**Table 1:** Descriptive Statistics for Measures.

				Frequency
Measure	Range	Mean	SD	(*n* out of 84)
Total father–child contact per week	0–9	2.13	2.81	
IFF-CG	0–38	24.0	13.0	
MSPSS-Family	4–28	20.0	7.0	
Employed prior to incarceration				50
Current jail stay offense: Child support				17
MAST				
no alcohol problems				44
borderline or alcohol problems				40
DAST				
no drug problem				39
drug problem				45

Note. IFF-CG = Inventory of Family Feelings-Father’s Ratings of Child’s Caregiver; MSPSS = Multidimensional Scale of Perceived Social Support; MAST = Michigan Alcoholism Screening Test; DAST = Drug Abuse Screening Test.

**Table 3:** Integrated Results Matrix for Qualitative and Quantitative Findings.

Theme	Qualitative Results	Example Quote	Quantitative Results	Integration of Results
Father as Provider for Child and Family	Fathers spoke about their desire to act as primary financial providers for their family.	“I want to get a job to get my family on my feet. Take them to the park. Play games with them”.	Paternal pre-incarceration employment related to increased odds of not recidivating in the first reentry year.	Fathers were motivated to work, provide for their families, and “treat” their children to positive experiences. Employment prior to incarceration may also make it easier to find a job following incarceration in jail, thus facilitating lower recidivism.
	Many fathers’ post-incarceration plans involved purchasing items for their children or taking them places.	“I’ll get released in a week, so that leaves me 20 days to get a job and a paycheck so I can get her a birthday present. If I can’t do that, it will be a real depressor”.		
Father as Nurturer of Child	Fathers spoke about their children as primary motivators for not reoffending.	“Just make up for lost time, you know? Never leave [my son] again hopefully”.	Frequency of father–child contact during incarceration was not directly related to reoffending in the first reentry year in the quantitative analyses. However, when fathers planned to live with the focal child after release, more father–child contact during incarceration related to less recidivism.	Fathers wanted to care for and nurture their children and one of the most difficult parts of incarceration was separation from their children. Children provide motivation for successful reentry. One way to care for and nurture children during incarceration was to have contact with them through visits and phone calls.
	Visits during incarceration helped some fathers stay connected to their children as they planned for release.	“I kind of know when I’m getting out so it’s a weight lifted off my shoulders. Now I have contact visits so it’s a lot better”.		
Father as Protector from the Criminal Justice System	Some fathers did not want their children to come to the jail to visit them because they were concerned about intergenerational cycles of criminal justice involvement.	“I don’t want [my son] here. When I was growing up I saw my dad through the glass”.	There was a significant correlation between the “father as protector” code and frequency of children’s visits to the jail; there was no correlation between “father as protector” and frequency of phone calls.	Fathers who were concerned about exposing their children to the carceral environment sacrificed seeing their children; however, many of them were able to talk on the phone to their children regularly instead.
	Some fathers did not want children to see them incarcerated because of stigma, shame, or wanting to better fulfill their role as father.	“Don’t want him to see me this way—I want him to be on a different path—I don’t want him to become comfortable with seeing me in jail”.		
Father–Child Contact and Family Support	For many fathers, making plans to rejoin their family’s daily routines was an important piece of their coping with their incarceration.	“Not being able to talk with her, tell her I love her. Not being able to make pancakes. Not being able to be a father one-hundred percent”.	When fathers planned to live with their child and also received average or high levels of family support, positive father–caregiver relationships were associated with high levels of father–child contact.	The ability of fathers to stay in touch with their young children during incarceration depended on children’s caregivers and fathers’ extended family. When fathers planned to live with their children during reentry, positive relationships with caregivers and family members were important. When fathers did not plan to live with their children during reentry, support from extended family helped them bypass caregivers’ gatekeeping roles.
	When talking about visits during incarceration, many fathers referenced the child’s mother acting as a gatekeeper between the father and child. This gatekeeping may be circumvented by other supports in the father’s family system (e.g., children’s grandmothers).	“I don’t know, it seemed like he wanted me and missed me. It’s always seemed like his mom didn’t want me to see him”.	When fathers did not plan to live with their children, father–caregiver relationship quality was unrelated to frequency of father–child contact, but family support predicted contact frequency.	

Note. We integrated quantitative data and qualitative data in this table to provide a more comprehensive description of our findings across different methods.

## Data Availability

The de-identified data set is available from the corresponding author upon request.

## Supplementary Materials

The following supporting information can be downloaded at: https://www.mdpi.com/1660-4601/19/6/3518/s1, Table S1: Nature of Fathers’ Offenses (N = 84).
