# Supplementary material for: “When She Says Daddy”: Black Fathers’ Recidivism following Reentry from Jail
Source: Int J Environ Res Public Health. 2022 Mar 16;19(6):3518. doi: 10.3390/ijerph19063518 (PMC8949043; doi:10.3390/ijerph19063518)
Supplement: Supplementary file 1 [file ijerph-19-03518-s001.zip › ijerph-1615955-supplementary.pdf]

Table S1 in Supplementary Materials

Nature of Fathers' Offenses (N=84).

Type of Offense	*n for Main Offense*	%	*n for Secondary Offense*	%
Probation technical violation/revocation	22	26.2	2	2.4
Non-payment of child support	15	17.9	2	2.4
Battery/violence	11	13.1	0	0
Possession of drugs	10	11.9	0	0
Disorderly conduct/domestic dispute	6	7.1	3	3.6
DUI/OWI	5	6.0	0	0
Theft/fraud/forgery	4	4.8	0	0
Property damage	5	6.0	5	6.0
Violence against an officer	2	2.4	4	4.8
No-contact order violation	1	1.2	1	1.2
Reckless endangerment of a child	1	1.2	0	0
Carrying a concealed weapon	1	1.2	0	0
Trespassing	1	1.2	0	0

Note. DUI = Driving under the influence, OWI = Operating while under the influence.
If more than one offense occurred, we list the more serious offense.
